# Nanoemulsification Improves the Pharmaceutical Properties and Bioactivities of Niaouli Essential Oil (*Melaleuca quinquenervia* L.)

**DOI:** 10.3390/molecules26164750

**Published:** 2021-08-05

**Authors:** Faiyaz Shakeel, Mounir M. Salem-Bekhit, Nazrul Haq, Sultan Alshehri

**Affiliations:** Department of Pharmaceutics, College of Pharmacy, King Saud University, Riyadh 11451, Saudi Arabia; faiyazs@fastmail.fm (F.S.); mbekhet@ksu.edu.sa (M.M.S.-B.); nazrulhaq59@gmail.com (N.H.)

**Keywords:** antiacne activity, *Melaleuca quinquenervia*, niaouli essential oil, nanoemulsification, phase diagrams, stability

## Abstract

We develop a suitable delivery system for niaouli essential oil (NEO) using a nanoemulsification method for acne vulgaris. Prepared nanoemulsions (NEs) were characterized for droplet dimension, rheology, surface charge, and stability. The ability of NEO formulations against *Propionibacterium acnes* and *Staphylococcus epidermidis* was investigated and all formulations showed antiacne potential in vitro. Ex vivo permeation studies indicated significant improvement in drug permeations and steady state flux of all NEO-NEs compared to the neat NEO (*p* < 0.05). On the basis of the studied pharmaceutical parameters, enhanced ex vivo skin permeation, and marked effect on acne pathogens, formulation NEO-NE_4_ was found to be the best (oil (NEO; 10% *v/v*); Kolliphor EL (9.25% *v/v*), Carbitol (27.75% *v/v*), and water (53% *v/v*)). Concisely, the in vitro and ex vivo results revealed that nanoemulsification improved the delivery as well as bioactivities of NEO significantly.

## 1. Introduction

The utilization of essential oils as active ingredients in food and pharmaceuticals is gaining massive impetus, both in terms of the increasing attention of consumers regarding active ingredients from natural resources and the increase in the concern regarding the adverse effects produced by synthetic compounds [1,2]. Due to their broad range of bioactivities and generally recognized as safe (GRAS) category status, several researchers have focused on identifying the phytotherapeutic efficacy of various plant-based essential oils in order to develop a safe product. Niaouli essential oil (NEO), obtained from *Melaleuca viridiflora* L. (Family: Myrtaceae), is native to northern Australia [3]. The functional component/biomarker compound of NEO has been reported as 1,8-cineole (around 55%), which may be responsible for the therapeutic activity of NEO, including in vitro antibacterial potential [3,4,5].

Topical drug delivery carriers have been established and appear to be very worthwhile for a number of drugs [6,7,8]. Topical drug delivery carriers offer several advantages over conventional routes of drug administration, especially when localized action is required [6]. In the last decade, the attention of several researchers has focused on nanoemulsion (NE) systems as alternative drug carriers to polymeric and lipid nanoparticles, as they combine many advantages and avoid most disadvantages associated with other colloidal drug carriers [8,9,10,11]. The NE technique is one the most studied techniques for enhancing the topical administration of essential oils with special regard to thermodynamic stability, protection of labile drugs from degradation, and excellent tolerability pattern [12,13,14,15].

Acne vulgaris is a common skin-inflammatory disorder of the sebaceous gland, which is due to the presence of *Propionibacterium acnes* (*P. acnes*) and *Staphylococcus epidermidis* (*S*. *epidermidis*), which cause irritation to the skin [16,17]. Therefore, the phytochemicals/drugs targeting acne vulgaris should be able to inhibit pathogens, reduce pro-inflammatory biomarkers, and to reduce post-acne scar development. Accordingly, the intended essential oils/phytochemicals aimed at acne treatment should have antibacterial and anti-inflammatory properties.

NEO has been investigated as a permeation enhancer for transdermal and cutaneous drug delivery of estradiol via gel formulations [18,19]. Various topical NE-based formulations of similar essential oils, such as tea tree oil, have been investigated for biological evaluation [20,21,22,23]. Topical NEs of other essential oils, such as eucalyptus essential oil, celery essential oil, psoralen, lemongrass essential oil, and babchi essential oil, have also been studied in the literature [12,24,25,26,27,28]. To the best of our knowledge, NEO has not been formulated in any topical NE formulation and there is no marketed product of NEO for effective acne treatment. Therefore, the aim of this research study was to optimize a topical NE-based drug delivery system emphasizing the topical administration of NEO in the management of acne vulgaris. The prepared NEs of NEO were characterized physicochemically and evaluated for physical stability, ex vivo skin permeation, and in vitro antiacne potential.

## 2. Results and Discussion

### 2.1. Pseudo-Ternary Phase Diagrams

**Figure 1:**
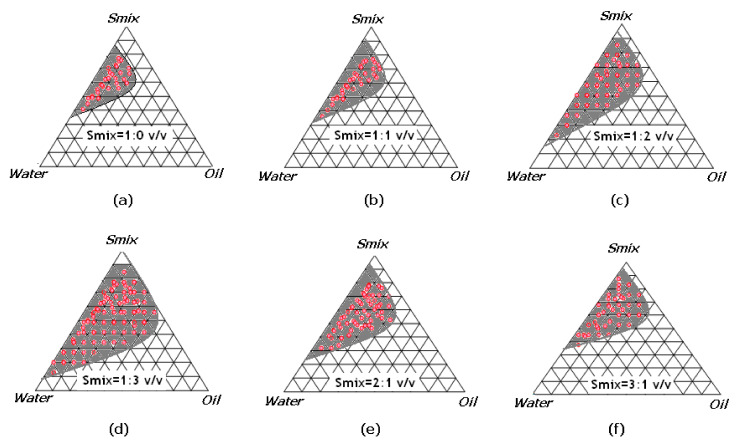
Nanophasic maps (shaded area) for oil (niouli essential oil (NEO)), surfactant (Kolliphor EL), and cosurfactant (Carbitol) at different S_mix_ ratios (Figure 1(**a**) (1:0), (**b**) (1:1), (**c**) (1:2), (**d**) (1:3), (**e**) (2:1), and (**f**) (3:1)).

Pseudo-ternary phase diagrams were established separately for each surfactant to cosurfactant (S_mix_) ratio and results are included in Figure 1a–f. From each phase diagram, oil-in-water (O/W) NE zones were delineated for the optimization of NEO-NEs. From the results presented in Figure 1, it was observed that the S_mix_ ratio of 1:0 (Figure 1a) was not strong enough to break the interfacial tension between the O/W interface. In this case, small NE zones were recorded along with more liquid crystalline (LC) phases in the phase diagram. However, in the case of the S_mix_ 1:1 ratio (Figure 1b), when a cosurfactant (Carbitol) was added to the surfactant (Kolliphor EL), the interfacial film was found to be more flexible and fewer LC phases were recorded compared to the S_mix_ 1:0. The largest amount of NEO (oil phase) which was solubilized was nearly 15.0 ± 1.85% *v/v* by utilizing S_mix_ of around 68.0 ± 4.34% *v/v* (Figure 1b). The amount of each component in NE was determined from each phase diagram using an aqueous phase titration method. These observations suggested the domination of surfactant (Kolliphor EL) in nanosizing in the presence of cosurfactant (Carbitol). It was observed that, when increasing the concentration of cosurfactant in the S_mix_ ratio (S_mix_ 1:2 and 1:3; Figure 1c,d), a marked decline in LC phases appeared with partial enhancement in the nanophasic region. However, on increasing the surfactant concentration in S_mix_ ratios (S_mix_ 2:1 and 3:1; Figure 1e,f), a significant increase in LC phases and scanty NE zones were recorded (*p* < 0.05). This observation could be due to a further drop in the interfacial tension and increase in the fluidity at the interface, leading to a slight increase in entropy of the system following more incorporation of oil in the hydrophobic region of the surfactant monomers [6,29]. The amplification in NE zones was insignificant when the surfactant concentration in S_mix_ was increased from 2:1 (Figure 1e) (*p* > 0.05). This could probably be an LC phase generated by Kolliphor EL and it was not removed with that concentration of cosurfactant.

### 2.2. Formulation Development

From each phase diagram, different NE formulations with different compositions were precisely selected. The entire NE zones in the phase diagrams were taken into consideration. Different formulation compositions having different oil concentrations with minimum possible S_mix_ concentrations were precisely selected from each phase diagram. The oil was taken and the required amount of S_mix_ was added in a prescribed ratio with drop-wise water addition till a clear and transparent formulation was obtained. The prepared NEO-NEs were tightly sealed in glass vials and stored at room temperature till further analysis. The compositions of these formulations are tabulated in Table 1.

### 2.3. Characterization and Optimization of NEs

#### 2.3.1. In Vitro Thermodynamic Stability Test

NEs are thermodynamically stable systems which are formed spontaneously [12,29]. In order to obtain stable formulations, the selected formulations were subjected to different thermodynamic tests. Those formulations, which were stable upon in vitro thermodynamic stability tests, were chosen for further optimization processes. The compositions of these formulations are depicted in Table 2. Formulations having <10% *v/v* of oil composition showed excellent transparency but the quantity of NEO was too low to accommodate therapeutic efficacy. This perception led to an increase the dose size of the formulation. Therefore, all the formulations having at least 10% *v/v* of oil composition were screened for the proposed study (Table 2).

#### 2.3.2. Droplet Diameter, Polydispersity Index (PDI), and Surface Charge Analysis

Table 3 summarizes the mean droplet diameter of selected formulations (NEO-NE_1_ to NEO-NE_6_) ranging from 95–162 nm. The largest droplet diameter appeared in NEO-NE_6_, which may have occurred due to the presence of a large amount of S_mix_ (Table 2). The droplet diameter analyses of the selected NEO-NEs indicated that the droplet diameter was increased with an increase in the NEO concentration and S_mix_ ratio. The PDI was recorded in the range of 0.252–0.819. It has been reported that the nanoformulations having a PDI in the range of 0.05–0.70 showed uniformity in their size distribution [30]. However, the nanoformulations having a PDI value of greater than 0.70 showed no uniformity in their size distribution. Formulation NEO-NE_4_ and NEO-NE_6_ have PDI values of greater than 0.70 and showed no uniformity in their size distribution. However, other formulations showed uniformity in their droplet size distribution. Formulation NEO-NE_4_ had the lowest PDI value (0.252), indicating the maximum uniformity in droplet diameter.

Generally, it has been reported that zeta potential values in the range of 25–30 mV characterize a stable formulation [31,32]. The zeta potential values of NEO-NEs were obtained in the range of 24.31–30.57 mV. Formulation NEO-NE_3_ had the highest zeta potential/surface charge (30.57 ± 3.72 mV). Overall, all formulations were found to be stable due to their values being in the range of 25–30 mV.

#### 2.3.3. Rheology

Table 3 summarizes the viscosity statistics of selected formulations (NEO-NE_1_ to NEO-NE_6_) ranging from 128–151 cp. The viscosity results can be correlated with the different contributions of Kolliphor EL as well as Carbitol utilized in the stabilization of selected NEs. The viscosity of the formulation NEO-NE_3_ was the lowest (128 cp) compared to the other NEs (NEO-NE_3_ < NEO-NE_4_ < NEO-NE_1_ < NEO-NE_2_ < NEO-NE_5_ < NEO-NE_6_), making it pertinent for topical use. The viscosity is dependent upon the S_mix_ ratio and also upon droplet diameter (providing a fixed quantity of the oil phase). Generally, the viscosity of each formulation was low due to the small droplet diameter. The obtained values of viscosity were suitable for the topical delivery of NEO and results were in good agreement with previous studies [12,33].

#### 2.3.4. Transmission Electron Microscopy (TEM) Analysis

**Figure 2:**
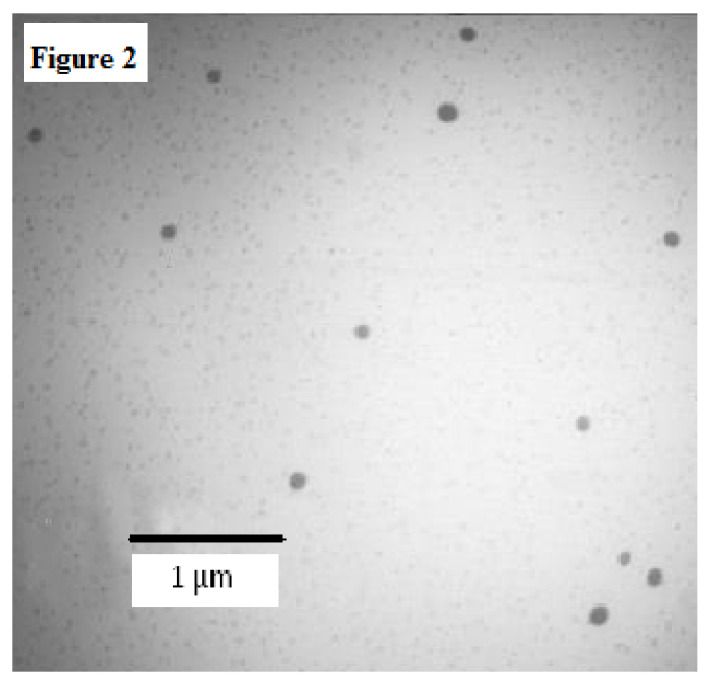
Transmission electron microscopy (TEM) image for an optimized formulation NEO-NE_4_.

The TEM images for an optimized formulation, NEO-NE_4_, were taken and interpreted for the droplet size distribution and surface morphology (Figure 2). The droplet diameter ranged from 95–162 nm and was in good agreement with droplet size, analyzed using a Zetasizer (Table 3). The shape of droplets was spherical and they were distributed in the nanometer range.

### 2.4. Ex Vivo Skin Permeation Studies

**Figure 3:**
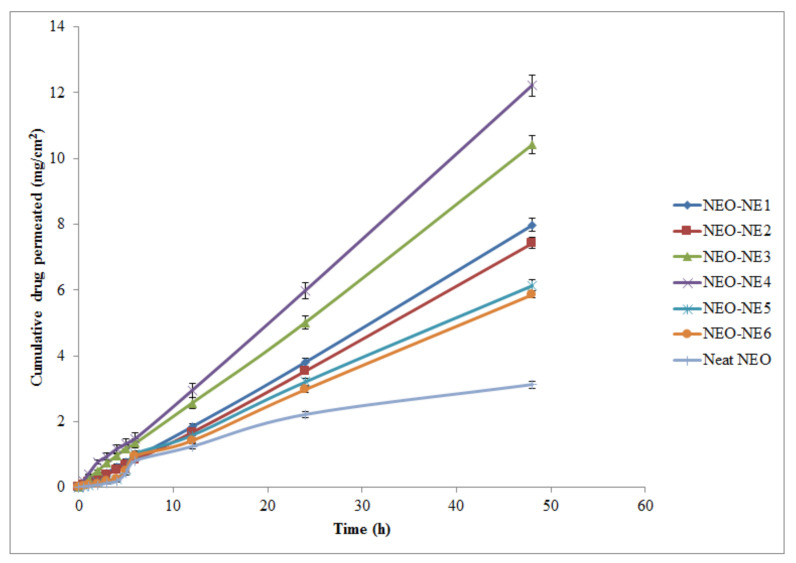
Ex vivo skin permeation profile of different nanoemulsions (NEO-NE_1_–NEO-NE_6_) and neat NEO (mean ± SD, *n* = 3.0).

Table 4 summarizes the details of ex vivo permeation evaluation. Formulation NEO-NE_4_ exhibited the highest permeation potential (cumulative NEO permeated); however, the lowest value was observed for neat NEO (Figure 3). The cumulative amounts of NEO permeated from formulation NEO-NE_4_ were 5.98 mg/cm^2^/h and 12.21 mg/cm^2^/h after 24 h and 48 h, respectively. Therefore, the clinically relevant dose for formulation NEO-NE_4_ was 10.52 mg and 21.48 mg for 24 h and 48 h of administration, respectively. The maximum permeation of formulation NEO-NE_4_ is perhaps due to its small droplet diameter and low viscosity as compared to other formulations. These results were found to be in good agreement with those reported for the skin permeation of caffeine, lemongrass oil, 5-fluorouracil, and hydrocortisone from NEs [9,12,33,34]. The probable mechanism by which NE formulations enhance the skin permeation of drugs is the lipid pathway of the stratum corneum, in which neutral lipids are arranged as bilayer structures with their hydrophobic chains facing each other to form a lipophilic bimolecular leaflet [32]. NEO, integrated in NE, directly penetrates into the stratum corneum, thereby destabilizing its bilayer structure with the help of surfactant present on the surface, leading to improved permeability of the lipid pathway to drug molecules. On the other hand, its hydrophilic domain deeply hydrates the stratum corneum, which is important in percutaneous drug uptake [17]. The small droplet diameter and low viscosity range that appeared in formulations suggest the NE system as an excellent carrier for the enhanced percutaneous uptake of NEO.

Various permeability parameters, such as *J*ss, *K*p, and *E*r, were significantly enhanced in NEO-NEs compared to the neat NEO (*p* < 0.05). The most perceptible grounds for the enhanced permeation of NEO from NEO-NE_4_ could be the combined effects of 1,8-cineole, Kolliphor EL, and Carbitol as well as the small droplet diameter and lowest viscosity.

### 2.5. Colloidal Stability Evaluation

All the selected formulations were kept in transparent glass vials and exposed to a stress-inducing temperature with a highly humid environment (40 °C/75% RH; regulated oven) for a minimum of three months to evaluate the influence of storage temperature on droplet diameter. A small augmentation in droplet diameter was observed in these storage conditions. The mean droplet diameter of the NEO-NE_4_ was increased from 95 nm to 112 nm in 90 days (Table 5). The results strongly support the protecting ability of Kolliphor EL in moderate temperature and humidity conditions. These results also agree with zeta potential data obtained in regulated conditions (Table 5). From the above findings, it can be seen that the droplet aggregation process accelerated with the increase in storage temperature. The small variations in droplet diameter and zeta potential after storage for 90 days suggested the stability of prepared NEO-NEs.

### 2.6. In Vitro Antiacne Study

The selected NEO-NEs exhibited antiacne activity against the aerobic microorganism *S. epidermidis* and anaerobic *P. acnes*. The antiacne activity, comparing MICs of neat NEO and NEO-NE formulations against the test organism, demonstrated that the NEO in formulation appears to retain its activity.

The effect of individual compounds utilized in making the NE formulations was screened to confirm their participation in the microbiological assay. Therefore, the surfactant (Kolliphor EL), cosurfactant (Carbitol), and neat NEO (5–10%), along with formulations (NEO-NE_1_–NEO-NE_6_) were assayed individually to compare their relative potential. NEs (NEO-NE_1_–NEO-NE_6_) exhibited higher antiacne effects than corresponding surfactant (Kolliphor EL), cosurfactant (Carbitol), and neat NEO itself, in the case of both organisms. Table 6 summarizes the inhibition potentials against both organisms. From Table 6, it is clear that NEO has an inhibitory role in the case of both pathogens. The role of the excipient in the assay has been shown but could be neglected. Neat NEO was used as a control, and concentrations ranging from 5–10% *v/v* in dimethyl sulfoxide (DMSO) were used to demonstrate the minimum bactericidal concentrations (MBCs) of oil. Formulation NEO-NE_4_ showed excellent inhibitory potential against both aerobic as well as anaerobic organisms. In addition, all formulations showed significant zone of inhibition values as compared to control (neat NEO), Kolliphor EL, and cosurfactant (Carbitol) (*p* < 0.05). The order of bioactivity for different formulations was NEO-NE_4_ > NEO-NE_3_ > NEO-NE_2_ > NEO-NE_5_ > NEO-NE_6_ > NEO-NE_1_. The maximum inhibitory potential for formulation NEO-NE_4_ may have been due to its small droplet diameter, which could have resulted in the largest contact surface and highest level of diffusion compared with the other formulations studied. These results were found in to be good agreement with those reported for the bioactivity of raloxifene hydrochloride, caffeine, and 5-fluorouracil from NEs [8,33,34].

## 3. Materials and Methods

### 3.1. Materials

NEO was obtained from the local market in Riyadh, Saudi Arabia. Carbitol, DMSO, and Kolliphor EL were procured from Sigma-Aldrich (St. Louis, MO, USA). Standard 1,8-cineole (purity: 99.6%) was purchased from Loba Chemicals (Bangalore, India). Water was obtained from a Milli-Q water purification system (Millipore, Billerica, MA, USA). For the antiacne assay, *Propionibacterium acnes* and *Staphylococcus epidermidis* were obtained from the American Type Culture Collection (ATCC, Manassas, VA, USA).

### 3.2. Analytical Methodology for the Analysis of NEO

**Figure 4:**
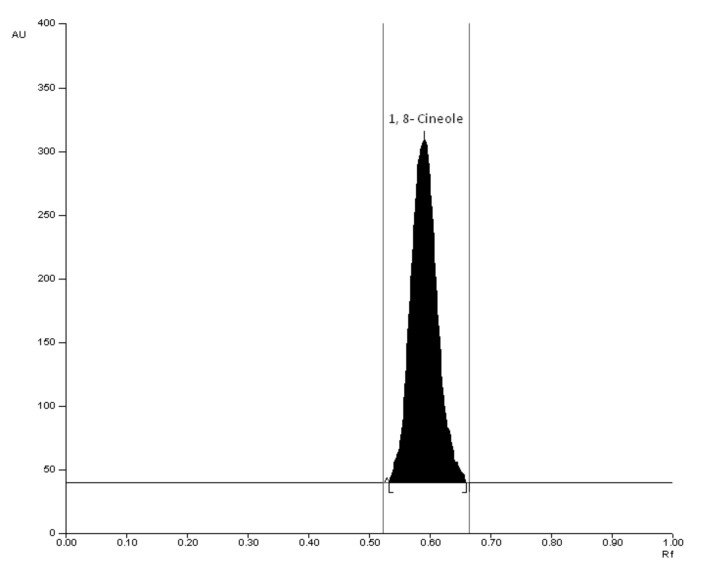
A typical chromatogram (R*F* = 0.59 ± 0.04) of 1,8-cineole (major constituent of NEO); mobile phase: toluene–ethyl acetate–formic acid (8.5:1.5:0.1, *v/v/v*).

NEO was analyzed by a validated high-performance thin-layer chromatographic (HPTLC) technique on the basis of 1,8-cineole, as it is the chief constituent of this oil (Figure 4). Chromatographic analysis was performed on 20 cm × 10 cm; linchosphere HPTLC plates pre-coated with 200 µm layers of silica gel 60 F_254_ (E-Merck, Darmstadt, Germany). The ternary combination of toluene:ethyl acetate:formic acid (8.5:1.5:0.1; *v/v/v*) was used as the mobile phase. A compact and well-resolved chromatogram of 1,8-cineole was obtained at retardation factor (*R_F_*) = 0.59 ± 0.04. The proposed HPTLC method for the quantification of 1,8-cineole was linear, accurate, precise, and robust. In addition, it was specific towards excipients. The quantification range of the proposed HPTLC method was 4-200 µg/mL. The regression equation was recorded as y = 98.576x + 404.24; in which x is the concentration of 1,8-cineole and y is the measured HPTLC response. The value of determination coefficient (R^2^) was determined as 0.9989. When NEO was investigated by the proposed HPTLC method for 1,8-cineole content, it was found to be approximately 54% *v/v*, suggesting the oil considered for preparation was authentic and of the best quality.

### 3.3. Construction of Pseudo-Ternary Phase Diagrams

For the development of an optimal concentration range of different components for the existence zone of NEs, pseudo-ternary phase diagrams were constructed using an aqueous titration technique [29]. For the screening of components for the development of NEs, a solubility study of the drug in different components is recommended [29,33,34]. The studied drug in the present study was NEO, which is miscible with surfactants and cosurfactants. Therefore, the solubility study was not performed. NEO was recommended as an oil phase without any incorporation of extra oil or combination. Kolliphor EL and Carbitol were randomly selected as surfactant and cosurfactant, respectively. Deionized water was used as the aqueous phase due to its frequent use in the literature [29]. The S_mix_ ratios were premixed in the volume ratios of 1:0, 1:1, 1:2, 1:3, 2:1, and 3:1. For each phase diagram, the oil phase (NEO) and a particular S_mix_ ratio were mixed thoroughly in the volume ratios of 1:9–9:1. Aqueous phase titration was performed for each S_mix_ ratio separately [9,29]. Visual observations were performed to identify the clear/transparent and easily flowable NE zones. Pseudo-ternary phase diagrams were constructed for each S_mix_ ratio and NE zones were marked on each phase diagram.

### 3.4. Formulation Development

Nanoemulsification was carried out for NEO (NEO-NEs), as reported in the literature [12]. Different formulations were prepared from each phase diagram. The required amount of oil phase (NEO) was first vortexed with the required amount of S_mix_ at 100 rpm at 25 ± 0.2 °C. The required amount of deionized water was then added drop by drop and vortexed in the same conditions till an apparent and clear solution was obtained.

### 3.5. Thermodynamic Stability Tests

In order to obtain the robust formulations and to remove metastable NEs, thermodynamic stability tests were conducted qualitatively [13,14]. Selected NEs were centrifuged using a Remi Centrifuge (model RM-1214, Mumbai, India) at 6000 rpm for 30 min and any physical changes, such as phase separation, creaming, or cracking, were observed. Formulations which were stable under centrifugation were further subjected to heating and cooling cycles (six cycles; temp: 0–45 °C; 48 h for either heating or cooling). Those formulations which survived the above two tests were subjected to freeze-pump thaw cycles (six cycles; temp: −21 °C to 25 °C; 48 h for either freeze or thaw). Visual observations were performed to observe any change in the consistency of NEs.

### 3.6. Droplet diameter, PDI, and Surface Charge Analysis

Droplet diameter, PDI, and surface charge of each formulation was determined using a Malvern Zetasizer (Nano ZS90; Malvern Instruments Ltd., Holtsville, UK). This method was based on the laser light scattering phenomenon, and analyzes the fluctuations due to the Brownian motion of droplets/particles. Light scattering was performed at 25 °C and a scattering angle of 90°. The samples of NEO-NEs (0.1 mL) were dispersed in 50 mL of deionized water and were used for analysis. The mean droplet diameter (Δ_dm_), PDI, and zeta potentials (*ζ*) were then recorded.

### 3.7. Rheology

The rheological behavior of each formulation was performed using a Brookfield DV III ultra V6.0 RV cone and plate viscometer (Brookfield Engineering Laboratories, Inc., Middleboro, MA, USA) using spindle # CPE40 at 25 ± 0.2 °C. All the rheological evaluations were performed at 25 ± 0.2 °C.

### 3.8. Refractive Index Determination

The refractive index (RI) of each NEO-NE formulation was determined using an Abbes type refractometer (Precision Testing Instruments Laboratory, Frankfurt, Germany) at 25 ± 0.1 °C [29].

### 3.9. TEM Analysis

The surface morphology of an optimized NEO-NE formulation was evaluated using a TEM (JEOL JEM 2100 F, Peabody, MA, USA). The TEM was capable of point to point resolution. The properly diluted samples (1:100 in deionized water) of an optimized formulation were used for the TEM analysis. A drop of diluted NEO-NE was deposited directly on a circular copper film grid and observed after drying without staining. The size and morphology were evaluated at different magnification modes [13].

### 3.10. Ex Vivo Skin Permeation Studies

Ex vivo skin permeation studies were carried out using a Franz diffusion cell with an effective diffusion surface area of 1.76 cm^2^ and 12 mL of receiver chamber capacity. Excised rat abdominal skin was used as the permeation membrane due to its easy availability. The Logan Transdermal Apparatus (SFDC6, Logan Instrument Corporation, Avalon, NJ, USA) was used to evaluate the ex vivo skin permeation profile of NEO-NEs. The excised skin was washed with deionized water and stored in a deep freezer at −21 °C till further evaluation. The skin was brought to room temperature at the time of experimentation. The skin was mounted between the donor and receiver compartment of the Franz diffusion cell in such a manner that the stratum corneum side faced the donor compartment and the dermal side faced the receiver compartment of the Franz diffusion cell. The skin was stabilized with permeation medium. For this, the receiver chamber was filled with ethanolic acetate buffer pH 5.4 (20:80 *v/v*) and stirred with a magnetic bead at a speed of 100 rpm in the Logan Transdermal apparatus at a temperature of 37 ± 1 °C. All buffer solution was replaced with fresh solution every 30 min in order to stabilize the skin. After running 12 cycles for stabilization, 0.5 mL of NEO-NE formulation was placed into the donor compartment which was equivalent to 27 mg of 1,8-cineole. The samples (0.5 mL from each batch) were withdrawn at regular intervals (0, 0.5, 1, 2, 3, 4, 5, 6, 12, 24, and 48 h) and replaced with freshly prepared drug-free permeation media. The samples were then filtered through a 0.45 μm membrane filter and subjected to the analysis of drug content directly using HPTLC at λ_max_ = 280 nm. The cumulative amount of drug permeated through the skin (μg/cm^2^) was plotted as a function of time (*T*) for each NEO-NE. The drug flux at a steady state (*J*_ss_) was calculated by dividing the slope of the linear portion of graph by the area of the Franz diffusion cell. The permeability coefficient (*K*_p_) for each NEO-NE was determined using Equation (1):

(1) $$K_{p}=\frac{J_{ss}}{C_{0}}$$

where C_0_ is the initial concentration of drug in the donor cell.

The enhancement ratio (*E*_r_) was determined using Equation (2):

(2) $$E_{r}=\frac{J_{ss}\;\left(formulation\right)}{J_{ss}\;\left(control\right)}\;$$

### 3.11. Colloidal Stability Evaluation

A short-term colloidal stability evaluation was carried out to observe the particle growth upon the storage of formulations (NEO-NE_1_–NEO-NE_6_) for a period of 90 days. Initially, the droplet size and zeta potential measurements were carried out for NEO-NEs. Further, they were divided into two batches and then kept in colorless and transparent glass vials under regulated temperature and humidity conditions (40 °C/75% RH) in a temperature-regulated oven under black boxes (light-protected conditions and avoiding interferences). The samples were taken out after 90 days for the droplet diameter and zeta potential analysis. NEs are well known as stable systems, both chemically and physically [35,36,37]. Therefore, only a physical/colloidal stability evaluation was performed in this work.

### 3.12. In Vitro Antiacne Study

NEO-NE formulations were evaluated for antiacne potential against surface organisms *S. epidermidis* and *P. acnes*. The study was carried out by using dehydrated brain heart infusion agar (BHIA) media prepared with 5% human blood in deionized water, maintaining a pH of 7.4 ± 0.2 (at 25 °C). Table 7 shows the BHIA medium composition with the components’ respective amounts. However, nutrient agar medium (NAM) was selected for the aerobic bacterium *S. epidermidis* assay.

The test organisms were maintained on the slants of medium and transferred to a fresh slant once/week. The microorganisms were washed from the agar slant on to a large agar surface (medium) and incubated for 48 h at 37 ± 1 °C. The growth from the nutrient surface was washed using 50 mL of deionized water and the test organisms were stored under refrigeration (4–8 °C). The method followed for the in vitro antiacne study was the cup and plate method [38]. A 200 µL amount of overnight nutrient broth culture of *S. epidermidis* was seeded into 20 mL of nutrient agar. The cups were bored using a sterile stainless steel borer. One Pasteur pipette drop of molten agar (45 °C) was added into the cups in order to seal the bottom. One hundred micrograms of NEO-NE formulations (NEO-NE_1_–NEO-NE_6_) containing 10% NEO were placed into cups and allowed to stand for 1 h and then incubated at 37 °C. Inhibition zone diameters were measured after 24 h of incubation. However, in the case of anaerobic organisms, one loopful of *P. acnes* was streaked out on 20 mL of BHIA medium. The cups were bored using a sterile stainless steel borer. One Pasteur pipette drop of molten agar (45 °C) was added into the cups in order to seal the bottom. NEO-NE formulations, ranging from NEO-NE_1_–NEO-NE_6_ containing 10% NEO were placed into cups and incubated in an anaerobic jar for 48 h. For control purposes, neat NEO (5–10%) prepared in dimethyl sulfoxide (DMSO) was considered. DMSO was used as a solvent for neat NEO and it was not present in formulations. Therefore, DMSO alone was not studied for bioactivity. After 48 h, the plates were examined and the zones of inhibition (mm) were accurately measured with a device [38,39].

### 3.13. Statistical Analysis

All the values are presented as mean ± SD of three observations. The statistical analysis was carried out by applying one-way analysis of variance (ANOVA) using the SPSS 11.0 program for Windows (Version 11.0, SPSS Co., San Diego, CA, USA). A significant difference was considered at a p value of less than 0.05.

## 4. Conclusions

Different NEs for NEO were developed using an aqueous phase titration technique. The prepared NEO-NEs were characterized well and evaluated for ex vivo skin permeation and in vitro antiacne potential. Based on maximum drug permeation, smallest droplet diameter, lowest PDI, lowest viscosity, optimum surfactant and cosurfactant concentration, and enhanced ex vivo skin permeation results, the formulation NEO-NE_4_ was considered as an optimized NE, containing oil (NEO; 10% *v/v*); Kolliphor EL 80 (9.25% *v/v*), Carbitol (27.75% *v/v*), and deionized water (53% *v/v*). From in vitro and ex vivo results, it can be concluded that the developed NE might be a promising carrier for improved topical delivery of NEO in the treatment of acne vulgaris. Further preclinical and clinical investigations are required to explore the complete potential of NEO-NEs.

## Figures and Tables

**Table 1:** Thermodynamic stability evaluation of obtained niaouli essential oil (NEO) nanoemulsions (NEO-NEs).

S_mix_ Ratio	Component (%)			Thermodynamic Stability Study			Inference
	Oil	S_mix_	Water	H/C	C/F	F/T	
1:0 (Figure 1a)	10	46	44	√	√	√	P
	10	50	40	√	-	√	F
	12	53	35	√	√	√	P
	12	55	33	√	-	-	F
	15	57	28	√	-	-	F
1:1 (Figure 1b)	10	36	54	√	√	√	P
	10	40	50	√	√	√	P
	12	37	51	√	√	√	P
	12	39	49	√	√	√	P
	15	37	48	-	-	-	F
1:2 (Figure 1c)	10	41	49	√	√	√	P
	10	33	57	√	√	√	P
	12	37	51	√	-	-	F
	12	43	45	√	√	√	P
	15	45	40	-	√	√	F
1:3 (Figure 1d)	10	36	54	√	-	√	F
	10	31	59	√	√	√	P
	12	41	49	√	√	√	P
	12	43	47	√	√	√	P
	15	44	41	-	-	-	F
2:1 (Figure 1e)	10	41	49	√	√	√	P
	10	46	44	√	√	√	P
	12	51	37	√	√	√	P
	12	49	39	√	√	√	P
	15	40	45	-	√	√	F
3:1 (Figure 1f)	10	46	44	√	√	√	P
	10	49	41	√	√	√	P
	12	53	35	√	√	√	P
	12	48	40	√	√	-	F
	15	55	30	√	√	X	F

H/C: Heating and cooling (0 °C and 45 °C); C/F: Centrifugation (6000 rpm); F/T: Freeze–thaw (−21 °C and +25 °C); P: Passed; F: Failed; -: Failed a particular test.

**Table 2:** Composition of selected NEO-NEs that passed thermodynamic stability tests.

Code	Formulation Composition (% *v/v*)			S_mix_ Combination
	Oil Phase	S_mix_ Phase	Water Phase	
NEO-NE_1_	10	46	44	1:0
NEO-NE_2_	10	36	54	1:1
NEO-NE_3_	10	33	57	1:2
NEO-NE_4_	10	31	59	1:3
NEO-NE_5_	10	41	49	2:1
NEO-NE_6_	10	46	44	3:1

**Table 3:** Droplet diameter, surface charge, and rheological measures of selected NEO-NEs (mean ± SD, *n* = 3).

Formulation	*Δ_dm_^#^* ± SD (nm)	*ζ*^§^ ± SD (mV)	*PDI* *	*R_I_*^†^ ± SD	*η*^$^ ± SD (cps)
NEO-NE_1_	136.52 ± 4.08	29.28 ± 2.68	0.731	1.452 ± 0.005	134.94 ± 8.19
NEO-NE_2_	138.61 ± 6.39	27.21 ± 1.98	0.648	1.460 ± 0.006	135.43 ± 7.41
NEO-NE_3_	119.70 ± 4.71	30.57 ± 3.72	0.357	1.457 ± 0.008	128.52 ± 9.18
NEO-NE_4_	95.86 ± 4.90	24.31 ± 4.81	0.252	1.453 ± 0.007	130.08 ± 6.87
NEO-NE_5_	158.08 ± 3.54	28.60 ± 5.76	0.758	1.451 ± 0.010	143.44 ± 8.72
NEO-NE_6_	162.41 ± 7.92	27.40 ± 3.91	0.819	1.468 ± 0.013	151.68 ± 6.07

*^#^* Average droplet diameter (Δ_dm_); * polydispersity index (PDI); ^§^ zeta potentials (*ζ*); ^†^ refractive index (*R_I_*); ^$^ viscosity mean (*η*); SD: standard deviation.

**Table 4:** Ex vivo permeability parameter of prepared NEs (mean ± SD, *n* = 3).

Matrices.	^#^*J*ss ± SD (mg/cm^2^/h)	^†^*K*p ± SD (cm/h ×10^−3^)	^¶^*E*r
* Neat NEO	0.0698 ± 0.0012	1.442 ± 0.2101	-
* NEO-NE_1_	0.1655 ± 0.0810	3.419 ± 0.2412	2.37
* NEO-NE_2_	0.1546 ± 0.0720	3.194 ± 0.2234	2.21
* NEO-NE_3_	0.2141 ± 0.0841	4.423 ± 0.3105	3.06
* NEO-NE_4_	0.2507 ± 0.0910	5.179 ± 0.4104	3.59
* NEO-NE_5_	0.1306 ± 0.0610	2.698 ± 0.2871	1.87
* NEO-NE_6_	0.1245 ± 0.0501	2.572 ± 0.2152	1.78

^#^*J*ss (Flux) = Cumulative amount of drug permeated/Time (mg/cm^2^/h). ^†^*K*p (Permeability coefficient) = Flux/Drug concentration (1,8-cineole) in donor cell. ^**¶**^*E*r (Enhancement ratio) = Flux of respective formulation/Flux of control formulation. * Dose: 0.5 mL of NEO-NE formulation (containing 27.5 mg of 1,8-cineole) was used for permeation.

**Table 5:** In vitro stability evaluation of NEs stored in regulated conditions (mean ± SD, *n* = 3).

Formulation Matrices	T_0_		90th Day Sampling	
			Stability Chamber (40 ± 2 °C/75 ± 5% RH) ^¶^	
	Δ_dm_ ± SD (nm)	*ζ* ± SD (mV)	Δ_dm_ ± SD (nm)	*ζ* ± SD (Mv)
NEO-NE_1_	136.52 ± 4.08	29.28 ± 2.68	151.22 ± 6.19	31.52 ± 4.96
NEO-NE_2_	138.61 ± 6.39	27.21 ± 1.98	149.31 ± 5.23	32.46 ± 3.80
NEO-NE_3_	119.70 ± 4.71	30.57 ± 3.72	127.57 ± 6.57	34.07 ± 2.47
NEO-NE_4_	95.86 ± 4.90	24.31 ± 4.81	112.19 ± 5.78	27.46 ± 3.21
NEO-NE_5_	158.08 ± 3.54	28.60 ± 5.76	173.44 ± 7.34	30.46 ± 3.48
NEO-NE_6_	162.41 ± 7.92	27.40 ± 3.91	180.72 ± 6.55	31.65 ± 4.73

^¶^ Accelerated stability testing of developed formulation (new dosage form) for minimum of 3 months as per ICH guidelines; Δ_dm_: Average particle diameter; *ζ*: Zeta potential; T_0_: Initial sampling.

**Table 6:** Antiacne assay of NEO-NEs.

Test Samples	Zone of Inhibition ^†^ (mm) ± SD	
	*S. epidermidis*	*P. acnes*
* Neat NEO (5% *v/v* in DMSO)	7.3 ± 0.2	4.1 ± 0.4
* Neat NEO (10% *v/v* in DMSO)	9.8 ± 0.5	6.9 ± 0.5
* Kolliphor EL	NA	NA
* Carbitol	3.4 ± 0.7	2.2 ± 0.7
* NEO-NE_1_	10.2 ± 0.5	7.1 ± 0.8
* NEO-NE_2_	13.5 ± 0.6	9.6 ± 0.6
* NEO-NE_3_	17.2 ± 0.6	11.8 ± 0.5
* NEO-NE_4_	20.4 ± 1.5	12.2 ± 0.2
* NEO-NE_5_	13.0 ± 0.3	7.5 ± 0.4
* NEO-NE_6_	12.8 ± 0.6	7.0 ± 1.5

^†^ Values of zone of inhibition (mm) ± SD; NA: No zone appeared. * An accurately measured amount of 100 mg (for each test sample) was placed in bored cups for assay.

**Table 7:** Brain heart infusion agar (BHIA) medium composition chart.

Ingredients	Quantity Required for 1000 mL (g)
Protease peptone	10.00
Calf brain infusion	200.00
Sodium chloride	5.00
Dextrose	2.00
Beef heart infusion	250.00
Agar	15.00
Disodium phosphate	2.50
Human blood	50.00

## Data Availability

This study did not report any data.

## Sample Availability

Samples of the NEO compounds are available from the authors.
